# Nontuberculous Mycobacterial Infection Mimicking Inflammatory Tenosynovitis: A Real Head Scratcher

**DOI:** 10.7759/cureus.20845

**Published:** 2021-12-31

**Authors:** Sambhawana Bhandari, Wendy Perdomo, Alla Rudinskaya, Karan Chawdhary, Oluwole Odujoko

**Affiliations:** 1 Internal Medicine, Danbury Hospital, Nuvance Health, Danbury, USA; 2 Rheumatology, Danbury Hospital, Nuvance Health, Danbury, USA; 3 Rheumatology, Wellspan Medical Group, York, USA; 4 Pathology and Laboratory Medicine, Danbury Hospital, Nuvance Health, Danbury, USA

**Keywords:** false-negative quantiferon, non-tuberculous mycobacterial infection hand, nontuberculous mycobacterial infection, tenosynovitis, mycobacteria

## Abstract

Nontuberculous mycobacterial tenosynovitis is a rare entity that is often misdiagnosed as bacterial or inflammatory tenosynovitis. We present a case of a 64-year-old man who presented with pain and swelling of his right wrist for several weeks. Magnetic resonance imaging (MRI) of his right upper extremity showed findings consistent with prominent tenosynovitis in the right extensor digitorum tendon sheath. Surgical debridement showed reactive histopathology with negative Gram stain, culture, and acid-fast bacilli stain; after which, steroids were started along with methotrexate and hydroxychloroquine, which was later changed to anti-tumor necrosis factor (anti-TNF) therapy. Due to minimal improvement, repeat operative debridement was done showing macroscopic rice bodies with pathology revealing chronic granulomatous inflammation with necrosis. However, repeated infectious work-up remained negative. After his symptoms progressed to involve his right index finger, his tenosynovium was sampled again, which was positive for acid-fast bacilli (AFB) staining for rare mycobacterial organisms, with cultures growing faint transparent colonies that were sent to the state laboratory for speciation. He was started on empiric therapy with clarithromycin, ethambutol, and rifampin following which his wound fully healed. This case illustrates the insidious course of nontuberculous mycobacteria (NTM) tenosynovitis leading to delayed diagnosis along with unwarranted treatments that could be harmful. Open tissue biopsy is important in the context of a lack of clinical response to common treatment modalities, in the absence of an alternative diagnosis with a similar clinical picture.

## Introduction

Nontuberculous mycobacteria (NTM) are ubiquitous organisms that rarely cause infection in immunocompetent individuals [1-3]. The primary site of involvement is the lungs, followed by lymph nodes (primarily cervical), and skin [3]. Musculoskeletal involvement by NTM infection is rare and the most commonly reported sites are the hands and wrists [1,2]. These infections are difficult to diagnose and are often misdiagnosed as bacterial or inflammatory tenosynovitis which often results in delayed or unwarranted treatments [1]. We report a case of NTM tenosynovitis of the hand which was misdiagnosed as inflammatory tenosynovitis and as such was treated with multiple anti-inflammatory and immunosuppressant medications without improvement.

## Case presentation

**Figure 1:**
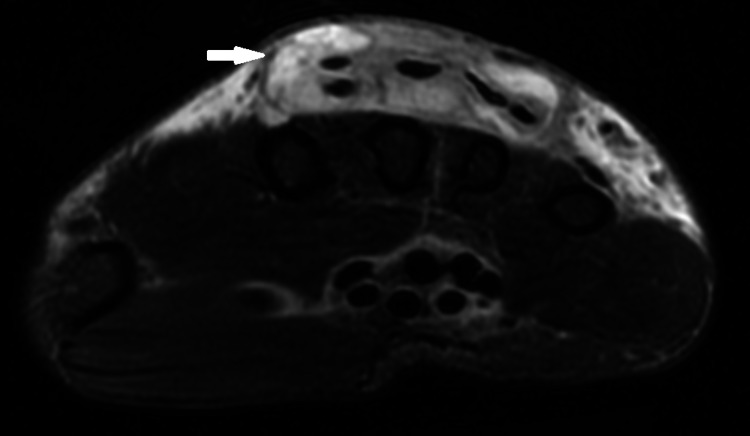
MRI of right upper extremity without contrast The image is showing abnormal tissue and edema within the sheath of the extensor digitorum, intermediate to high signal in T2-weighted images, consistent with prominent tenosynovitis.

**Figure 2:**
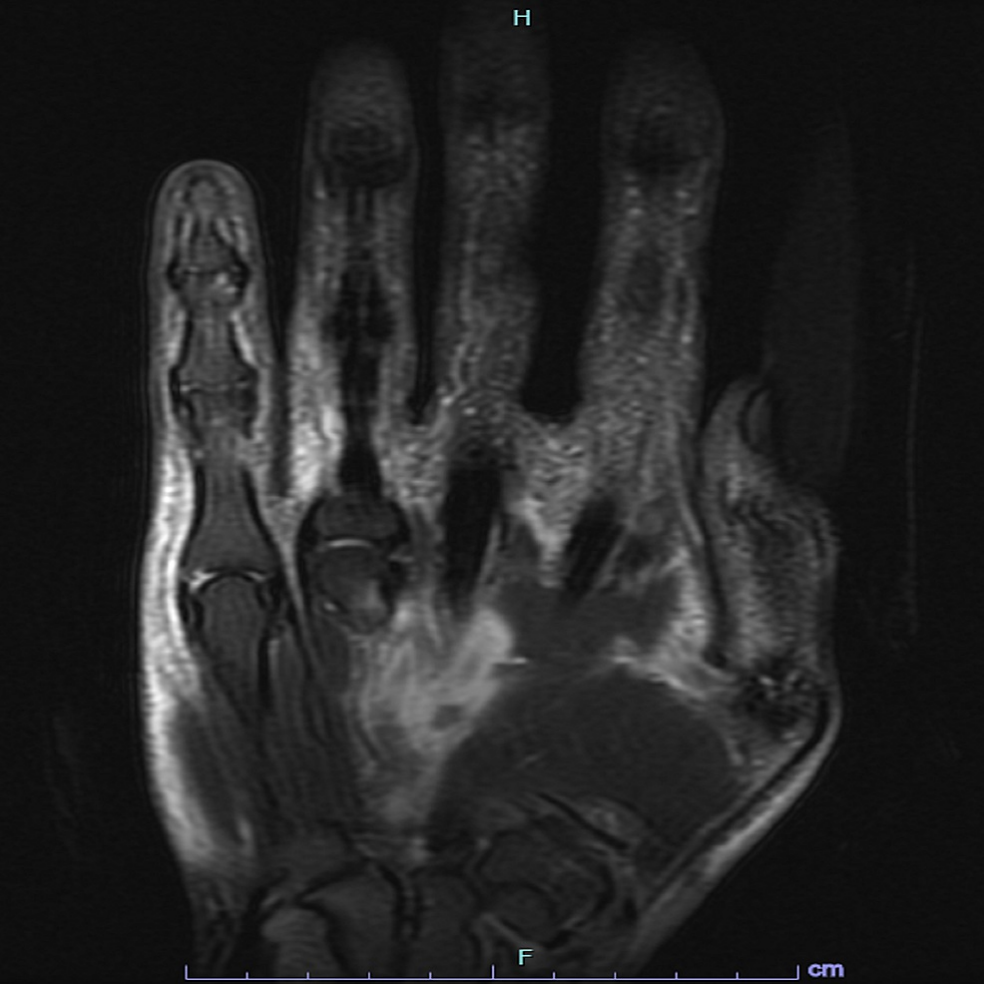
MRI of the right wrist without contrast The image is showing dorsal subcutaneous edema throughout the hand and engulfing the dorsal tendon sheaths.

A 64-year-old male presented with pain, swelling, and stiffness on his right hand and wrist for several weeks. He had an intravenous line placed for a colonoscopy (done for screening purposes), a couple of weeks prior to the onset of symptoms. He denied any other previous trauma, surgical procedures, or intravenous drug use. He denied fever, chills, shortness of breath, or abdominal pain. There was no history of psoriasis/rheumatoid arthritis or involvement of other joints. Physical examination revealed tenderness and extensive swelling of the dorsum of the right hand and wrist without overlying erythema or nail changes. Initial workup was negative for rheumatoid factor, anti-nuclear antibody (ANA), and tick panel with normal erythrocyte sedimentation rate (ESR), and C-reactive protein (CRP). Synovial fluid analysis from the right wrist joint showed 7000 leukocytes and was negative for gram stain, culture, and crystals. X-ray of the right hand didn't show any acute findings. At this point, there was a suspicion of soft tissue infection involving the right hand/wrist, and magnetic resonance imaging (MRI) was done. Magnetic resonance imaging (MRI) of the right wrist without contrast showed prominent dorsal subcutaneous edema around the wrists, distal forearm, and proximal hand with abnormal tissue within the sheath of the extensor digitorum, manifesting an intermediate to high signal on T2-weighted images, consistent with prominent tenosynovitis in the right extensor digitorum tendon sheaths (Figures 1, 2).

**Figure 3:**
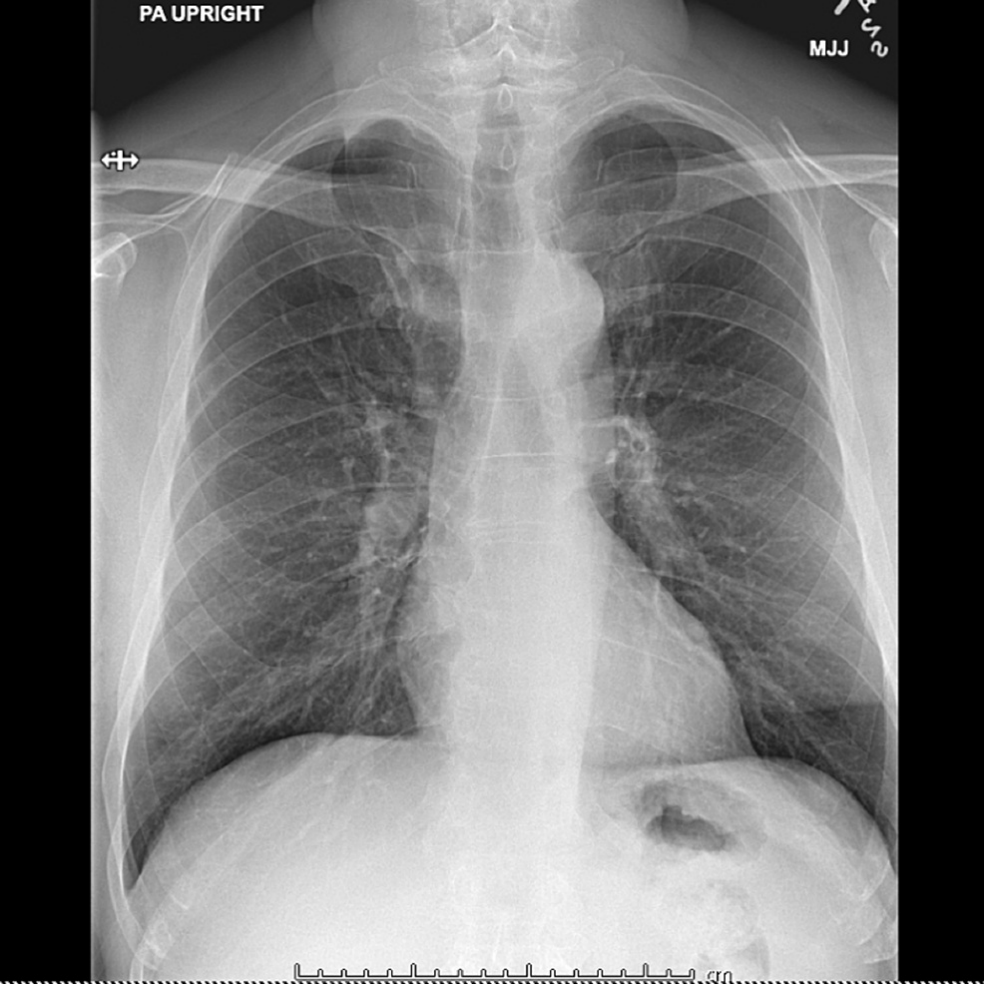
Chest x-ray of the patient shows hyperinflation without any other abnormalities

He underwent surgical debridement with a tissue diagnosis positive for reactive histopathology with negative Gram stain, culture, and acid-fast bacilli stain. With the working diagnosis of inflammatory tenosynovitis of unclear etiology, he was started on steroids along with methotrexate and hydroxychloroquine for several months. Due to persistent symptoms, he was started on biological therapy with anti-tumor necrosis factor (anti-TNF) after a negative QuantiFERON test and a normal chest x-ray (Figure 3).

**Figure 4:**
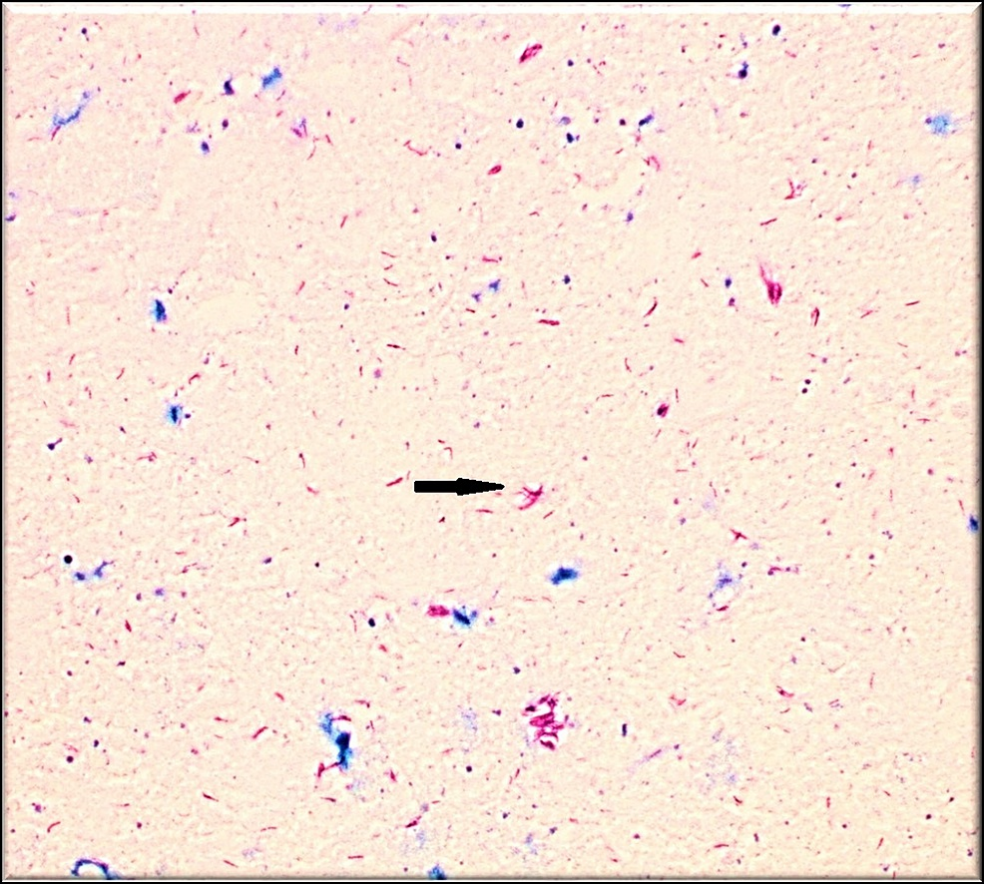
Positive acid-fast bacilli stain - right index finger The black arrow shows a positive acid-fast bacilli stain.

**Figure 5:**
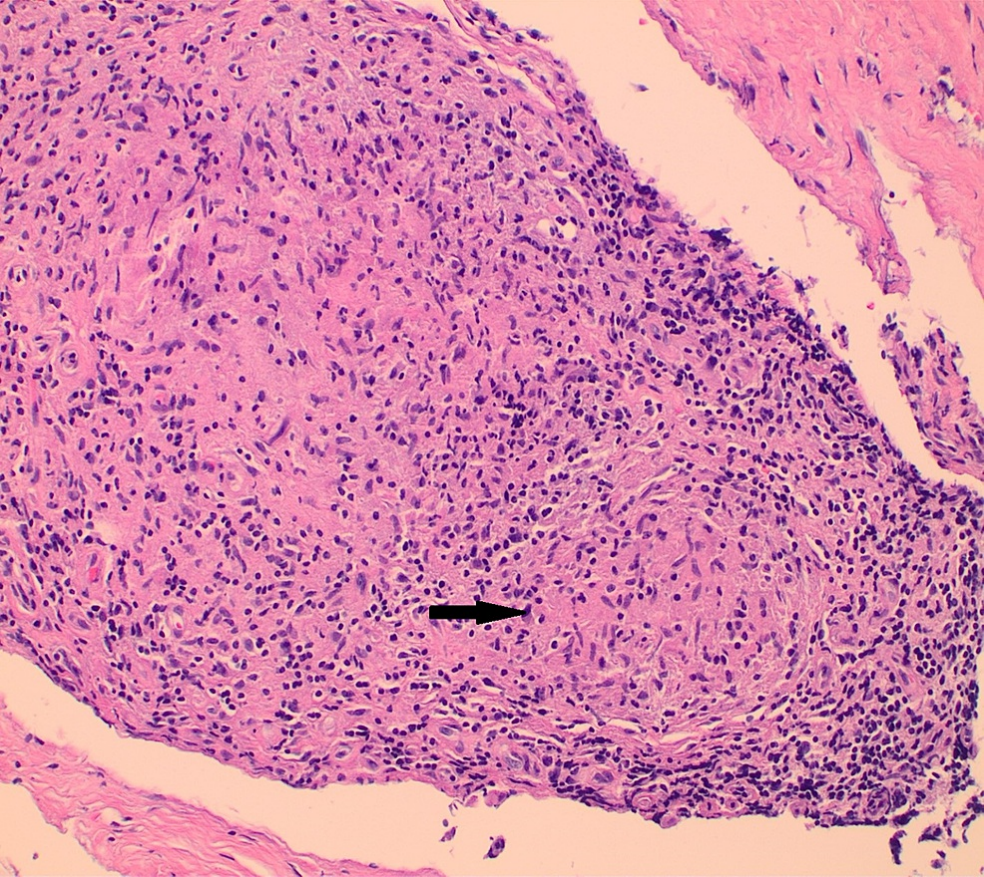
Hematoxylin-eosin stain showing granulomas with histiocytes and peripheral mononuclear lymphocytes The black arrow shows a granuloma with histiocytes and peripheral rim of lymphocytes.

Despite these therapies, he only expressed minimal improvement in his right wrist pain and swelling. An MRI was repeated showing persistent findings as mentioned above. He then underwent a repeat operative debridement which surprisingly showed macroscopic rice bodies with pathology revealing chronic granulomatous inflammation with necrosis. The tissue was again sent for infectious workup as mentioned above, which continued to remain negative. His symptoms progressed to involve his right index finger, after which his tenosynovium was sampled again and histopathology revealed chronic tenosynovitis with non-necrotizing granulomas, with positive AFB stain for rare mycobacterial organisms (Figures 4, 5).

The patient was then referred to an infectious disease specialist. He then had repeated debridement of his right hand and wrist with positive AFB stain and cultures that grew intermediate to slow-growing, fastidious mycobacterial species that were sent to the state lab for speciation. He was started on empiric therapy with clarithromycin, ethambutol, and rifampin. After further debridement, his wound was fully closed, symptoms improved, and the decision was made to continue with antimicrobial therapy for 12-18 months after closure. Speciation wasn’t able to be done even at the state laboratory.

## Discussion

Musculoskeletal involvement by NTM is very rare; its incidence is reported to be about 5-10%, with hands and wrists being the most frequently involved sites [1]. Musculoskeletal or extrapulmonary infections are usually seen in immunosuppressed individuals and are very rare in immunocompetent individuals [3]. The most commonly involved causative agents are *Mycobacterium marinum* and *Mycobacterium kansasii* [1]. In our particular case, cultures grew slow-growing fastidious mycobacteria, which could include other uncommon possibilities such as *Mycobacterium avium* complex, *M. kansasii*, *M. marinum*, *Mycobacterium ulcerans*, *Mycobacterium haemophilum*, *Mycobacterium xenopi*, *Mycobacterium simiae*, and *Mycobacterium malmoense* [4]. Among the pathogens most commonly involved, *M. marinum* is found primarily in contaminated water, putting fishermen and aquarium cleaners at greater risk [1,4]. The known modes of transmission include the history of trauma, surgical procedures, intravenous drug use, dermal piercings, and hematogenous (usually from the lungs).

These infections are indolent in contrast to pyogenic bacterial infections which lead to delay in the diagnosis leading to an increase in morbidity [5]. Imaging modalities are nonspecific both in the early and late stages and could resemble other reactive/inflammatory/granulomatous pathologies [2]. An open biopsy followed by specific cultures is the most reliable way of diagnosis [1,4]. Rice bodies are small fibrin bodies that can be seen with chronic synovium inflammation, rheumatoid arthritis, tubercular mycobacterial infection, atypical mycobacterial infection, and osteoarthritis to name a few. In our case, the rice bodies involved extensor tendon sheaths and joint spaces were spared, which was more in favor of NTM/tubercular tenosynovitis [1,5].

Some mycobacterial species have very specific growth requirements that lead to false-negative cultures. For example, *M. ulcerans* requires a low oxygen concentration and a temperature between 29°C and 33°C to grow, making it very cumbersome for diagnosis [4]. A QuantiFERON test was performed before starting anti-TNF therapy in our patient, which was negative. The rate of positive results for different organisms varies, which have been reported to be 1% for *M. avium*, 1% for *Mycobacterium intracellulare*, 52% for *M. kansasii*, 58% for *M. marinum*, 33% for *Mycobacterium szulgai*, 0% for *Mycobacterium abscessus*, and 0% for *Mycobacterium chelonae *[6]. Therefore, a low threshold for suspicion with repeated tissue diagnosis, including AFB stain and culture, is needed for diagnosis as presented in this case. Debridement is equally essential along with the adequate duration of combination medical therapy for appropriate treatment and wound healing [4,5].

## Conclusions

This case illustrates the insidious course of NTM tenosynovitis leading to delayed diagnosis along with unwarranted treatments, that could be harmful. Musculoskeletal involvement is possible in the absence of lung involvement, and repeatedly negative QuantiFERON test, chest x-ray, and acid-fast bacilli (AFB) stains from synovial fluid do not rule out this infection. Open tissue biopsy is very important if there is a lack of response to common modalities of treatment, especially in the absence of an alternative diagnosis.
